# The Intention of Hiccup

**Published:** 1849-01

**Authors:** 


*The Intention of Hiccup.*—In the convulsive movement of hiccup, the diaphragm is depressed; the larynx is raised; and the glottis is closed. What would be the effects of these conditions? The depression of the diaphragm would tend to expand the cavity of the chest; but the glottis being closed, no air can enter the lungs. The two ends of the œsophagus are, however, still open, and if the hiccup be strong enough, air will enter the œsophagus at both ends. If a person will make a prolonged voluntary effort of the conditions which occur in hiccup, he will find a portion of air sucked, as it were, into the œsophagus, from the pharynx. Now, spasmodic hiccup is a reflex movement, excited, in general, by gaseous irritation of the stomach; under these conditions the hiccup will suck the air of the stomach into the lower extremity of the œsophagus. This, then, is the intention of hiccup—to pump off the air of the stomach. The movement of the hiccup sucks the gaseous contents of the stomach into the lower extremity of the œsophagus, and an inverted action of the œsophagus propels them upwards, and discharges them at the pharynx.—*Prov. Med. and Surg. Jour.*

